# Wearable Sensors Measure Ankle Joint Changes of Patients with Parkinson's Disease before and after Acute Levodopa Challenge

**DOI:** 10.1155/2020/2976535

**Published:** 2020-04-09

**Authors:** Zhuang Wu, Xu Jiang, Min Zhong, Bo Shen, Jun Zhu, Yang Pan, Jingde Dong, Pingyi Xu, Wenbin Zhang, Li Zhang

**Affiliations:** ^1^Department of Geriatrics, Affiliated Brain Hospital of Nanjing Medical University, Nanjing, China; ^2^Department of Neurology, First Affiliated Hospital of Guangzhou Medical University, Guangzhou, China; ^3^Department of Neurosurgery, Affiliated Brain Hospital of Nanjing Medical University, Nanjing, China

## Abstract

**Background:**

Previous studies found levodopa could improve the activity of the ankle joints of patients with Parkinson's disease (PD). But ankle joint movement is composed of four motion ranges. The specific changes of four motion ranges in PD remain unknown.

**Objective:**

The purpose of this study was to decompose the complex ankle joint movement, measure ankle joint changes before and after the acute levodopa challenge test (ALCT), and investigate the effects of these parameters on gait performance.

**Methods:**

29 PD patients and 30 healthy control subjects (HC) completed the Instrumented Stand and Walk (ISAW) test and gait parameters were collected by the JiBuEn gait analysis system. The percentage of improvement of gait data and the UPDRS III in the on-drug condition (ON) were determined with respect to the off-drug condition (OFF).

**Results:**

We observed a reduction in the heel strike angle (HS), 3-plantarflexion (3-PF) angle, and 4-dorsiflexion (4-DF) angle of ankle joints. We did not find significant difference in the toe-off angle (TO), 1-plantarflexion (1-PF) angle, and 2-dorsiflexion (2-DF) angle among three groups. Stride length improvement rate was significantly correlated with HS (*r*_s_ = 0.616, *P* < 0.001) and 3-PF (*r*_s_ = 0.639, *P* < 0.001) improvement rates. The improvement in the sum of rigidity items (UPDRS motor subsection item 22) was also correlated with HS (*r*_s_ = 0.389, *P*=0.037) and 3-PF (*r*_s_ = 0.373, *P*=0.046) improvement rates.

**Conclusions:**

Exogenous levodopa supplementation can significantly reduce the rigidity of patients with PD, improve their 3-PF and 4-DF of ankle joint kinematic parameters, and ultimately enhance their gait.

## 1. Introduction

Parkinson's disease (PD) is the second most common neurodegenerative disease. Gait and balance impairments are important clinical characteristics of patients with PD [1]. These patients exhibit slow turns, small step length, and arm swing [2]. With disease exacerbation, their gait disturbance may induce falls and fractures, which increases mortality [3]. Therefore, gait intervention is essential in these patients. Supplementation with exogenous levodopa is suggested to improve motor function in patients with PD. Levodopa intake can improve stride length and velocity [4–7]. In addition, the peak velocity of arm during walking, stride duration, double support time, cadence, and trunk movement can also be improved [4, 8]. However, most studies focused on spatiotemporal gait parameters which are insufficient to elucidate complex gait characteristics in patients with PD [9, 10]. The changes of these spatiotemporal gait parameters in patients with PD remain controversial. An increasing number of studies have focused on kinematic parameters, such as joint movements which may shed more light on improvement mechanisms [11]. Patients with cerebellar ataxia have increased step width and reduced ankle joint range which makes it possible to distinguish cerebellar ataxia from the healthy group [12]. Levodopa can improve the activity of the hip, knee, and ankle joints of patients with PD [13, 14]. After levodopa intake, only the kinematic parameters of ankle joints show increased regularity [15]. Therefore, the ankle joint is of great significance for patients with PD. However, ankle joint movement is complicated and is composed of four motion ranges with corresponding functions [16–18]. A previous study has demonstrated that exogenous levodopa supplementation can significantly reduce the rigidity of patients with PD [19]. We propose that with the improvement of rigidity of patients with PD, it can release some kinematic parameters of ankle joints and ultimately enhance their gait. With the development of microelectronics technology, wearable sensors can objectively and quantitatively assess the walking function of the human body [20–22]. In our study, we collected data by using the latest wearable device, the JiBuEn gait analysis system [23]. The purpose of this study was to decompose the complex ankle joint movement, measure ankle joint changes before and after the ALCT, and investigate the effects of these parameters on gait performance. These parameters may be used to effectively elucidate the mechanism of levodopa on gait improvement and evaluate the effect of pharmacological treatments.

## 2. Patients and Methods

### 2.1. Patients

In our study, 29 patients with PD (18 men, 11 women; mean duration of disease 6.4 ± 4.6 years) were enrolled in the Department of Geriatrics, the Affiliated Brain Hospital of Nanjing Medical University, between October 2018 and July 2019. All patients with idiopathic PD were diagnosed according to the Movement Disorder Society (MDS) criteria [24] and were free of neurological or musculoskeletal disease that may affect the results of our study. We recruited 30 HC subjects from the spouses or caregivers of the patients with PD. After complete explanation of the study, all participants signed written informed consent prior to the experiment. This research was approved by the Medical Ethics Committee of the Affiliated Brain Hospital of Nanjing Medical University, and the IRB approval number was “2017-KY037.”

### 2.2. Clinical Evaluation

For both PD patients and HC subjects, we collected the following data: age, height, weight, gender, shoe size, and Montreal Cognitive Assessment (MoCA). All patients were tested in the morning and were instructed to fast before the ALCT. Their antiparkinsonian medication was stopped for at least 24 h (72 h for controlled release antiparkinsonian medication). The state of the patients at this time was defined as the OFF. We then conducted the first Unified Parkinson's Disease Rating Scale (UPDRS) assessment. After the first assessment, the patients were administered with levodopa at 1.5 times their regular morning dose. After approximately 1 h, the patients were asked to describe their subjective feelings on their levodopa intake. When they felt the best response such as relaxed, it was defined as ON [13]. We conducted the second UPDRS assessment. Two UPDRS assessments for each patient with PD were independently assessed by two neurology specialists, and the final results were averaged.

**Figure 1:**
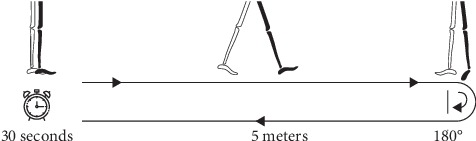
All the participants performed Instrumented Stand and Walk (ISAW) test: they were asked to stand quietly for 30 seconds with their arms at their sides and look straight ahead, walk 5 meters, turn 180 degrees after crossing a line on the ground, and return to the initial starting position. The test was performed twice before and after the ALCT for the patients with PD and only once for HC.

All participants completed the Instrumented Stand and Walk (ISAW) test [25] (Figure 1) which was a reliable and sensitive measure of gait. ISAW consisted of standing quietly for 30 seconds with their arms at their sides and look straight ahead, followed by a verbal instruction to initiate gait, walk 5 meters, turn 180 degrees after crossing a line on the ground, and return to the initial starting position. We explained the procedures of ISAW in detail to all participants before the test. In addition, all participants walked twice in advance to be familiar with the test. This test was performed twice before and after the ALCT by the patients with PD and only once for HC.

### 2.3. Equipment

The JiBuEn gait analysis system was used to collect gait data [23]. The system includes modules with inertial microelectromechanical system sensors and Bluetooth module fixed under the smart shoes. In addition, four external modules were attached to the patient's calf and thigh (the upper and lower sides of the knee joint). These modules collected motion signals and transmitted them to computer. The system uses a high-order low-pass filter and hexahedral calibration technique in data preprocessing. This calibration technique can reduce high-frequency noise and installation errors produced by the sensor devices. In addition, based on the zero-correction algorithm, the accumulative errors can also be corrected. By using the quaternary complementary filtering technique, the final gait parameters are obtained by fusing acceleration data and posture. By the JiBuEn gait analysis system, we can collect spatiotemporal gait parameters (total steps of ISAW, stride length, stride time, gait velocity, cadence, stride time, swing phase time, and stance phase time) and kinematic parameters (heel strike angle, toe-off angle, plantarflexion angle, and dorsiflexion angle of ankle joints).

### 2.4. Statistical Analysis

Data were expressed as mean ± SD. For all analyses, a *P* value <0.05 was regarded as significant. For clinical characteristics and gait parameters of both groups, quantitative data were initially analyzed with the Kolmogorov–Smirnov test to check for whether the data follow normal distribution. Two-sample *t*-test was performed to compare the differences between PD patients and HC subjects when both sets of data followed normal distribution, otherwise the Mann–Whitney *U* test was used. Paired *t* test was performed to compare the differences between the OFF and ON states in PD patients when both sets of data followed normal distribution; otherwise, Wilcoxon signed-ranks test was performed. Chi-square test was used for qualitative data. The associations between UPDRS assessment scores and gait data were explored using Spearman correlation analysis. All data were analyzed using the IBM SPSS software version 23. Figures were configured using Graph Pad Prism Software version 8.0.1.

## 3. Results

To compare our research with previous studies, we also measured the spatiotemporal gait parameters of gait. All data were presented in the following parts.

### 3.1. Clinical Characteristics of Participants

Fifty-nine participants joined this study, and their clinical characteristics are shown in Table 1. No statistical difference was found for all baseline data. The mean duration of disease was 6.4 ± 4.6 years. The mean Hoehn–Yahr (H-Y) stage of the disease was 2.7 ± 0.7. The total UPDRS III scored 35.8 ± 16.9 in the OFF state, and UPDRS III improvement rate was 39.8% ± 15.3%.

### 3.2. Changes in Spatiotemporal Gait Parameters after Levodopa Intake

**Figure 2:**
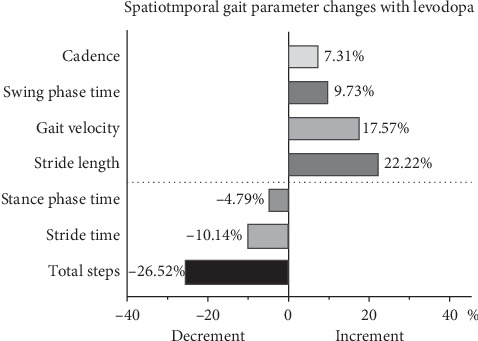
Spatiotemporal gait parameters changes with levodopa. The data was shown in %.

We measured spatiotemporal gait parameters, including total steps of ISAW, stride length, gait velocity, cadence, stride time, stance phase time, and swing phase time. We observed significant differences among the three groups in these spatiotemporal gait parameters (Table 2). We then calculated the rate of changes for each parameter in patients with PD (Figure 2). The total number of steps for patients with PD was 18.1 ± 9.7 steps in the OFF state, which was significantly reduced by ∼26.52% in the ON state. Compared with the OFF state, the stride length increased significantly by ∼22.22% in the ON state in patients with PD. We also observed increased gait velocity, cadence, and swing phase time and decreased stride time and stance phase time after levodopa intake.

### 3.3. Changes in Foot Joint Angle Parameters after Levodopa Intake

**Figure 3:**
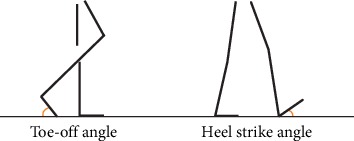
Toe-off angle and heel strike angle of the right leg.

**Figure 4:**
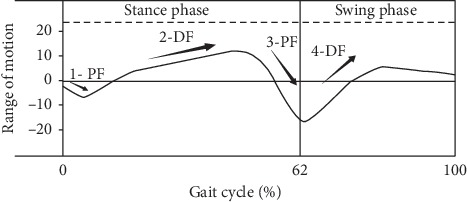
The ankle joint walking patterns of normal men. Each gait cycle of normal men includes stance phase and swing phase. The ankle joint moves through four ranges of motion, alternating between plantarflexion (PF) and dorsiflexion (DF). Among them, the first three motion ranges (1-PF, 2-DF, and 3-PF) occur in the stance phase, and the fourth (4-DF) occurs in the swing phase.

**Figure 5:**
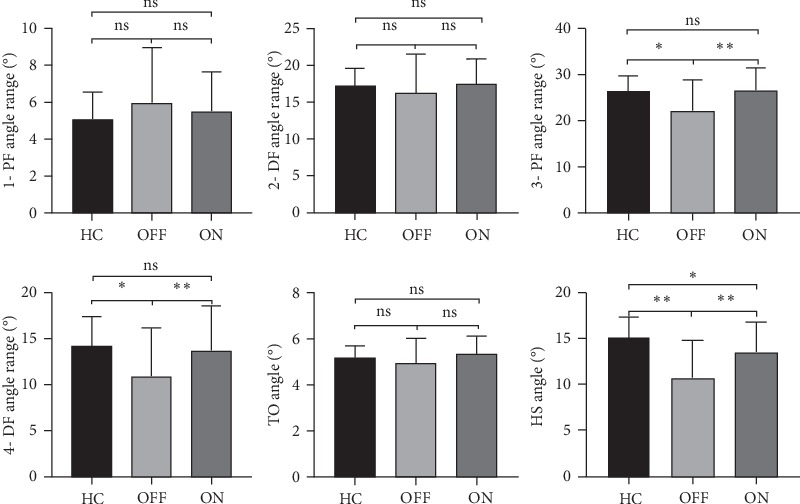
Wearable sensors measure ankle joint changes. 1-PF, 2-DF, 3-PF, and 4-DF angles and TO and HS angles are depicted according to groups. Group data are displayed as mean ± SD. PF, plantarflexion; DF, dorsiflexion; TO angle, toe-off angle; HS angle, heel strike angle. “ns” means no significance, ^*∗*^*P* < 0.05, ^*∗∗*^*P* < 0.001.

Using the latest JiBuEn gait analysis system, we measured six parameters of foot joints: TO, HS (Figure 3), two plantarflexion (PF) angles, and two dorsiflexion (DF) angles of ankle joints (Figure 4). These angles are small, but they are essential for normal gait. We observed significant differences in HS, 3-PF, and 4-DF among the three groups. Especially for 3-PF and 4-DF, great significant differences were found between the OFF and ON states but not between the HC and ON states. This finding suggested the important mechanism of levodopa. However, we observed no significant difference in TO, 1-PF, and 2-DF among three groups (Figure 5).

### 3.4. Correlation between Objectively and Subjectively Acquired Motor Improvement Rate

We calculated the improvement rate of gait parameters and items of UPDRS III before and after levodopa intake. Stride length improvement rate was significantly correlated with foot joint kinematic parameter improvement rate. The improvement in the sum of rigidity items (UPDRS motor subsection item 22) was also correlated with the foot joint angle parameter improvement rate (Table 3).

## 4. Discussion

Our trial involving 29 patients with PD and 30 healthy adults indicated that exogenous levodopa supplementation could significantly improve the spatiotemporal gait parameters and ankle joint kinematic parameters of patients with PD. We collected the kinematic parameters of patients during walking by using a wearable device. For comparison with previous studies, we also collected spatiotemporal gait parameters.

For spatiotemporal gait parameters, the new gait analysis system showed smaller step length and slower gait velocity in patients with PD compared with those in HC. These changes reflect the characteristics of “bradykinesia” in these patients [26]. Note that cadence has no significant differences between PD and HC, indicating that slow gait is primarily due to the small step length rather than the small cadence [27]. In patients with PD, cadence was improved to a low extent in the ON state, and this finding is concordant with a previous study [4]. A great increment in stride length, gait velocity, cadence, and swing phase time was found after levodopa intake, whereas a decrement in stride time, total steps, and stance phase time was observed in the ON state. Patients with PD also felt a great improvement, such as feeling relaxed, when asked to walk during their ON state. Our study suggests that levodopa can significantly improve the spatiotemporal gait parameters of PD patients, and this finding is consistent with results of previous studies [2, 4, 20, 28].

For the kinematic parameters, our research expands the study on ankle joints in patients with PD. The movements of the ankle joint mainly include dorsiflexion (DF) and plantarflexion (PF). Although these angles are small, each angle has its unique function and is essential for normal walking. Our findings confirmed those of a previous study on gait alternations in HS and TO angles, in which a significant reduction was found in HS but not in TO [29]. The results of correlation analysis suggest that HS is gradually decreasing as the disease progresses. Similar to small gait, shuffling is also an important feature of the gait of patients with PD. The decrease in HS indicates increased difficulty in lifting the foot, which may result in falls. However, we found statistical difference between the ON state and HC. These results indicate that levodopa can only improve HS to some extent. We found that TO does not change in PD.

In each gait cycle, the ankle joint moves through four ranges of motion, alternating between PF and DF. Among them, the first three angles (1-PF, 2-DF, and 3-PF) occur in the stance phase, whereas the fourth (4-DF) occurs in the swing phase. The average ankle motion range is approximately 25° in each gait cycle, which is consistent with our results [16, 17]. 1-PF and 2-DF revealed no changes. However, 3-PF and 4-DF were significantly decreased in patients with PD. No difference was found between HC and ON, suggesting the important mechanism of levodopa. In the normal gait cycle, the heel first hits the ground. At this time, the weight of the body is also quickly transferred to the heel, leading to the first rapid plantarflexion angle of the ankle joint–1-PF [30]. This action can slow down the speed of the lower limbs, thus reducing the impact of rapid body sudden falls [31]. 2-DF runs through the entire stance phase, during which the body completes transferring the center of body mass [31]. Sufficient 2-DF is essential for pulling the shin bone to move the body forward. In the swing phrase, 2-DF is also the driving force for advancement [32]. Our results suggest that the motor performance of patients with PD was not different from that of HC in these two angles. This finding indicates that patients with PD do not have an impaired ability to delay the effect of rapid body sudden falls and to transfer the center of body mass. This finding conflicts with that of previous studies, stating that patients with PD have a low ability to transfer the center of body mass [33]. We tentatively interpret this result to difference of disease duration. The average disease duration of the patients with PD in the present study is 6.4 years which is shorter than that in a previous study. With disease progression, the movement disorder gradually worsens, and stability is influenced. In addition, the present study only focused on ankle joint movement. 3-PF occurs in the early stage of the swing phase to prepare the hind foot for the swing phase. In our study, we found that the 3-PF of patients with PD was significantly reduced, suggesting their insufficient connection of gait cycle. However, 3-PF was not statistically different between the ON state and HC. The force outburst of 3-PF is the rebound of the soleus and gastrocnemius muscles after relaxation [34, 35]. We attributed this finding to the effect of levodopa on rigidity improvement. 4-DF, which is beneficial for lifting the foot off the ground to complete the clearance and promote the advancement of the lower limb, occurs at the beginning of the swing phase. Similar to 3-PF, the 4-DF of patients with PD was significantly reduced. Consistent with a latest study, the ankle joint muscles appear to be weak in patients with PD [36]. A reduced 4-DF may result in falls. The decreased rigidity may have possibly increased the 3-PF and 4-DF after levodopa intake.

Axial rigidity in patients with PD during turning is associated with extra turning steps [10]. Patients with PD have a decrement of stride length which may be attributed to a decreased sagittal inclination angle [37]. In patients with PD, ankle PF is associated with gait velocity and stride length [38]. These articles all suggested that kinematic parameters had an impact on spatiotemporal gait parameters. However, in the complex ankle joint movement, it is unknown that kinematic parameter will affect the gait of patients with PD. There were six kinematic parameters of the foot joint in our study. Only three parameters, heel strike angle (HS), 3-plantarflexion angle (3-PF), and 4-dorsiflexion angle (4-DF), were impaired. Compared with that on the OFF state, the stride length was significantly increased by∼22.22% in the ON state in patients with PD. Therefore, we performed correlation analysis for the improvement of stride length and these three kinematic parameters, respectively. The results indicated that the improvement of HS and 3-PF were significantly correlated with the improvement of stride length. In summary, we have reason to propose that the improvement of the HS and 3-PF made contributions to the improvement of the spatiotemporal gait parameter, stride length. Levodopa is a basic treatment for patients with PD, and rigidity shows the best response to L-dopa [39]. The rigidity items of UPDRS III were improved after exogenous levodopa intake, resulting in the improved kinematic parameters.

Our research and previous studies confirmed that patients developed significantly improved gait performance after exogenous levodopa supplementation. The strengths of our study include restrictive enrollment criteria, a set of new and accurate gait research equipment, focusing on the analysis of kinematic parameters, decomposing complex ankle joint movements and the use of subjective and objective measures of gait characteristics. The findings may have pragmatic implications for pharmacological and rehabilitation treatments. Levodopa has become the mainstay therapy for PD and is beneficial for the treatment of motor symptoms. However, it cannot relieve all kinematic parameters, such as HS in our study, and thus requires further research to complement this impairment.

Our study also has some limitations. First, walking requires the coordinated movement of joints and muscles throughout the entire body. However, our research only focused on the ankle joint. Joint movement is complicated and cannot be fully explained in an article, and the function of ankle joint is of great importance for patients with PD [13, 15]. Second, the mean H-Y stage of patients with PD is 2.7 ± 0.7. Our study did not include patients with late stage disease because they cannot walk independently under the OFF state. Finally, only 29 PD patients with PD were enrolled, causing difficulty in drawing general conclusions. Further research must focus on larger sample sizes and different disease stages.

## 5. Conclusion

The decrement of ankle joint mobility in patients with PD is mainly manifested in the HS, 3-PF, and 4-DF angles instead of TO, 1-PF, and 2-DF angles. Exogenous levodopa administration can improve 3-PF and 4-DF angles. However, it can only improve HS to some extent. By reducing the rigidity of patients with PD, exogenous levodopa supplementation can significantly improve their 3-PF and 4-DF of ankle joint kinematic parameters and ultimately enhance their gait.

## Figures and Tables

**Table 1:** Clinical characteristics of participants.

	HC	PD	*P*
N	30	29	
Age	62.7 ± 6.7	65.7 ± 10.2	0.185
Height (cm)	164 ± 4.9	167 ± 8.0	0.180
Weight (kg)	61.8 ± 9.6	66.2 ± 9.5	0.082
BMI (M)	22.9	23.8	0.111
Male (%)	16 (53.3)	18 (62.1)	0.497
Shoe size (M)	7	8	0.060
MoCA	24.4 ± 0.8	24.0 ± 1.1	0.080
Duration of PD (years)		6.4 ± 4.6	
Hoehn–Yahr stage		2.7 ± 0.7	
UPDRS III total scores in OFF state		35.8 ± 16.9	
UPDRS III improvement scores		15.1 ± 9.9	
UPDRS III improvement rate (%)		39.8 ± 15.3	

Data are shown as mean ± SD and median. M: median. BMI: body mass index. MoCA: montreal cognitive assessment. UPDRSIII: Unified Parkinson's disease rating scale part 3.

**Table 2:** Spatiotemporal gait parameters of participants.

	Groups			*P*		
	HC	OFF	ON	HC vs OFF	HC vs ON	OFF vs ON
Total steps (steps)	10.6 ± 2.1	18.1 ± 9.7	13.3 ± 5.8	<0.001^*∗∗*^	0.002^*∗*^	<0.001^*∗∗*^
Stride length (m)	1.2 ± 0.1	0.9 ± 0.3	1.1 ± 0.2	<0.001^*∗∗*^	0.009^*∗*^	<0.001^*∗∗*^
Gait velocity (m/s)	0.91 ± 0.1	0.74 ± 0.3	0.87 ± 0.2	0.004^*∗*^	0.414	<0.001^*∗∗*^
Cadence (steps/min)	93.8 ± 9.5	91.6 ± 19	98.3 ± 12.0	0.564	0.120	0.048^*∗*^
Stride time (s)	1.29 ± 0.1	1.38 ± 0.34	1.24 ± 0.16	0.229	0.192	0.028^*∗*^
Stance phase time (%)	64.87 ± 2.21	67.01 ± 6.69	63.80 ± 3.43	0.617	0.115	0.013^*∗*^
Swing phase time (%)	35.13 ± 2.22	32.99 ± 6.71	36.20 ± 3.43	0.622	0.120	0.012^*∗*^

Spatiotemporal gait parameters of participants. Data are shown as mean ± SD. ^*∗*^*P* < 0.05; ^*∗∗*^*P* < 0.001.

**Table 3:** Correlation between objectively and subjectively acquired motor improvement in PD patients.

	*r*_s_	*P*
H-Y stage vs OFF HS	−0.419	0.024^*∗*^
H-Y stage vs OFF 3-PF	−0.287	0.131
H-Y stage vs OFF 4-DF	−0.229	0.231
Stride length improvement vs HS improvement	0.616	<0.001^*∗∗*^
Stride length improvement vs 3-PF improvement	0.639	<0.001^*∗∗*^
Stride length improvement vs 4-DF improvement	0.357	0.058
Rigidity item improvement vs HS improvement	0.389	0.037^*∗*^
Rigidity item improvement vs 3-PF improvement	0.373	0.046^*∗*^
Rigidity item improvement vs 4-DF improvement	0.141	0.465

H–Y stage: Hoehn–Yahr stage. Rigidity items: UPDRS motor subsection item 22. HS: heel strike angle. 3-PF: 3-plantarflexion angle of ankle joints. 4-DF: 4-dorsiflexion angle of ankle joints. ^*∗*^*P* < 0.05; ^*∗∗*^*P* < 0.001.

## Data Availability

The data used to support the findings of this study are available from the corresponding author upon request.

## References

[1] Pistacchi M., Gioulis M., Sanson F. (2017). Gait analysis and clinical correlations in early Parkinson’s disease. *Functional Neurology*.

[2] Smulders K., Dale M. L., Carlson-Kuhta P., Nutt J. G., Horak F. B. (2016). Pharmacological treatment in Parkinson’s disease: effects on gait. *Parkinsonism & Related Disorders*.

[3] Avanzino L., Lagravinese G., Abbruzzese G., Pelosin E. (2018). Relationships between gait and emotion in Parkinson’s disease: a narrative review. *Gait & Posture*.

[4] Curtze C., Nutt J. G., Carlson-Kuhta P., Mancini M., Horak F. B. (2015). Levodopa is a double-edged sword for balance and gait in people with Parkinson’s disease. *Movement Disorders*.

[5] Bryant M. S., Rintala D. H., Hou J. G. (2011). Gait variability in Parkinson’s disease: influence of walking speed and dopaminergic treatment. *Neurological Research*.

[6] Rochester L., Baker K., Nieuwboer A., Burn D. (2011). Targeting dopa-sensitive and dopa-resistant gait dysfunction in Parkinson’s disease: selective responses to internal and external cues. *Movement Disorders*.

[7] Fregni F., Boggio P. S., Bermpohl F. (2006). Immediate placebo effect in Parkinson’s disease—is the subjective relief accompanied by objective improvement?. *European Neurology*.

[8] Sterling N. W., Cusumano J. P., Shaham N. (2015). Dopaminergic modulation of arm swing during gait among Parkinson’s disease patients. *Journal of Parkinson’s Disease*.

[9] Park K., Roemmich R. T., Elrod J. M., Hass C. J., Hsiao-Wecksler E. T. (2016). Effects of aging and Parkinson’s disease on joint coupling, symmetry, complexity and variability of lower limb movements during gait. *Clinical Biomechanics*.

[10] Yang W.-C., Hsu W.-L., Wu R.-M., Lu T.-W., Lin K.-H. (2016). Motion analysis of axial rotation and gait stability during turning in people with Parkinson’s disease. *Gait & Posture*.

[11] Kuhman D., Hammond K. G., Hurt C. P. (2018). Altered joint kinetic strategies of healthy older adults and individuals with Parkinson’s disease to walk at faster speeds. *Journal of Biomechanics*.

[12] Serrao M., Chini G., Bergantino M. (2018). Identification of specific gait patterns in patients with cerebellar ataxia, spastic paraplegia, and Parkinson’s disease: a non-hierarchical cluster analysis. *Human Movement Science*.

[13] Foreman K. B., Singer M. L., Addison O., Marcus R. L., LaStayo P. C., Dibble L. E. (2014). Effects of dopamine replacement therapy on lower extremity kinetics and kinematics during a rapid force production task in persons with Parkinson disease. *Gait & Posture*.

[14] Robichaud J. A., Pfann K. D., Comella C. L., Corcos D. M. (2002). Effect of medication on EMG patterns in individuals with Parkinson’s disease. *Movement Disorders*.

[15] Kurz M. J., Hou J. G. (2010). Levodopa influences the regularity of the ankle joint kinematics in individuals with Parkinson’s disease. *Journal of Computational Neuroscience*.

[16] Cerny K., Perry J., Walker J. M. (1990). Effect of an unrestricted knee-ankle-foot orthosis on the stance phase of gait in healthy persons. *Orthopedics*.

[17] Kadaba M. P., Ramakrishnan H. K., Wootten M. E., Gainey J., Gorton G., Cochran G. V. B. (1989). Repeatability of kinematic, kinetic, and electromyographic data in normal adult gait. *Journal of Orthopaedic Research*.

[18] Verdini F., Marcucci M., Benedetti M. G., Leo T. (2006). Identification and characterisation of heel strike transient. *Gait & Posture*.

[19] Birkmayer W., Hornykiewicz O. (1961). The L-3, 4-dioxyphenylalanine (DOPA)-effect in Parkinson-akinesia. *Wien Klin Wochenschr*.

[20] Horak F. B., Mancini M. (2013). Objective biomarkers of balance and gait for Parkinson’s disease using body-worn sensors. *Movement Disorders*.

[21] Mohamed Refai M. I., van Beijnum B.-J. F., Buurke J. H., Veltink P. H. (2019). Gait and dynamic balance sensing using wearable foot sensors. *IEEE Transactions on Neural Systems and Rehabilitation Engineering*.

[22] Dobkin B. H., Martinez C. (2018). Wearable sensors to monitor, enable feedback, and measure outcomes of activity and practice. *Current Neurology and Neuroscience Reports*.

[23] Tao S., Zhang X., Cai H., Lv Z., Hu C., Xie H. (2018). Gait based biometric personal authentication by using MEMS inertial sensors. *Journal of Ambient Intelligence and Humanized Computing*.

[24] Postuma R. B., Berg D., Stern M. (2015). MDS clinical diagnostic criteria for Parkinson’s disease. *Movement Disorders*.

[25] Mancini M., King L., Salarian A., Holmstrom L., McNames J., Horak F. B. (2011). Mobility lab to assess balance and gait with synchronized body-worn sensors. *Journal of Bioengineering and Biomedical Science*.

[26] Schilder J. C. M., Overmars S. S., Marinus J., van Hilten J. J., Koehler P. J. (2017). The terminology of akinesia, bradykinesia and hypokinesia: past, present and future. *Parkinsonism & Related Disorders*.

[27] Bowes S., Clark P., Leeman A. (1990). Determinants of gait in the elderly parkinsonian on maintenance levodopa/carbidopa therapy. *British Journal of Clinical Pharmacology*.

[28] Rabel C., Le Goff F., Lefaucheur R. (2016). Subjective perceived motor improvement after acute levodopa challenge in Parkinson’s disease. *Journal of Parkinson’s Disease*.

[29] Schlachetzki J. C. M., Barth J., Marxreiter F. (2017). Wearable sensors objectively measure gait parameters in Parkinson’s disease. *PLoS One*.

[30] Maganaris C. N. (2001). Force-length characteristics of in vivo human skeletal muscle. *Acta Physiologica Scandinavica*.

[31] Maganaris C. N. (2002). Tensile properties of in vivo human tendinous tissue. *Journal of Biomechanics*.

[32] Maganaris C. N., Paul J. P. (2000). Hysteresis measurements in intact human tendon. *Journal of Biomechanics*.

[33] Mancini M., Rocchi L., Horak F. B., Chiari L. (2008). Effects of Parkinson’s disease and levodopa on functional limits of stability. *Clinical Biomechanics*.

[34] Ishikawa M., Komi P. V., Grey M. J., Lepola V., Bruggemann G.-P. (2005). Muscle-tendon interaction and elastic energy usage in human walking. *Journal of Applied Physiology*.

[35] Bojsen-Moller J., Hansen P., Aagaard P., Svantesson U., Kjaer M., Magnusson S. P. (2004). Differential displacement of the human soleus and medial gastrocnemius aponeuroses during isometric plantar flexor contractions in vivo. *Journal of Applied Physiology*.

[36] Skinner J. W., Christou E. A., Hass C. J. (2019). Lower extremity muscle strength and force variability in persons with Parkinson disease. *Journal of Neurologic Physical Therapy*.

[37] Huang S.-C., Lu T.-W., Chen H.-L., Wang T.-M., Chou L.-S. (2008). Age and height effects on the center of mass and center of pressure inclination angles during obstacle-crossing. *Medical Engineering & Physics*.

[38] Rafferty M. R., Prodoehl J., Robichaud J. A. (2017). Effects of 2 Years of exercise on gait impairment in people with Parkinson disease. *Journal of Neurologic Physical Therapy*.

[39] Sethi K. (2008). Levodopa unresponsive symptoms in Parkinson disease. *Movement Disorders*.

